# Case of Compound Fracture of the Ankle Joints

**Published:** 1807-08

**Authors:** 

**Affiliations:** Ketley


### CASE *of* COMPOUND FRACTURE *of the* ANKLE JOINTS; *By* MR. EVANS, *of Ketley.*

JULY 1, 1806. James Dainty, a boy 15 years of age, was with several other persons descending into a coal mine by means of a rope fastened to the barrel of a steam engine, which being under the management of a careless boy, part of the machinery gave way, and they were all precipitated to the bottom of the pit, by which accident he had both his legs fractured. The right had a compound fracture of the ankle joint, with a wound 9 inches long in a curved direction up the limb, and the astragalus completely removed from its connexion with the os calcis and metatarsal bones, lying out of the joint, adhering to a portion of the capsular ligament with the foot turned inwards, exposing the whole cavity to view. From the appearance of such a formidable injury, it was a matter of some consideration whether it would be most prudent to amputate or attempt the preservation of the limb; from my usual success in the cure of compound fractures, where the principal blood vessels were *not* divided, I was inclined to adopt the latter plan. After the astragalus was separated from the portion of ligament to which it was attached, the wound was cleared by means of a sponge well moistened with warm water, and the foot placed in its natural position, and the edges of the wound brought in contact with sutures; the parts were afterward covered with soft linen cloth of several folds, well saturated with a solution of crude ammonia in vinegar, over which a bandage was applied to secure the foot in its proper situation; the leg was then laid on its outside, with the knee bent, supported by pillows; the bandage being kept constantly wet with the above solution. By the 27th of the same month the tension had subsided, and the discharge from the wound greatly diminished; the cavity of the joint being filled with healthy granulations, which gradually became more firm. In the space of four months the patient was able to bear some weight upon the limb, the wound being three parts cicatrized. In nine months he was perfectly cured, and returned to his work without any deformity. He enjoys some degree of motion in the joint, and walks with little lameness at this time.

N. B. The bone is now in my possession for the inspection of any one who may wish to see it.

*July* 13, 1807.

